# Effects of a compassion-based program on the grief experienced by caregivers of people suffering from dementia: a randomized controlled clinical trial

**DOI:** 10.1186/s12912-022-00980-5

**Published:** 2022-07-25

**Authors:** Leila Jahani, Shahla Abolhassani, Sima Babaee, Victoria Omranifard

**Affiliations:** 1 Science of Medical and Surgical Nursing, Faculty of Nursing and Midwifery, Isfahan University of Medical Sciences, Isfahan, Iran; 2 Adults Health Nursing Department, Faculty of Nursing and Midwifery, Nursing and Midwifery Care Research Center, Isfahan University of Medical Sciences, No. 81746-73461, Hezar Jerib Road, Isfahan, Iran; 3 Department of Psychiatry, Behavioral Sciences Research Center, Isfahan University of Medical Sciences, Isfahan, Iran

**Keywords:** Dementia, Caregiver, Empathy, Grief

## Abstract

**Background:**

Dementia is a syndrome that reduces the cognitive and functional abilities of the brain increasing the need for care. The caregivers of these patients are mostly their family members. The great care burden causes devastating effects on the health of family caregivers and the grief experienced by these family caregivers is considerable. This study was conducted to investigate the effects of a compassion-based program on the grief experienced by caregivers of people suffering from dementia.

**Method:**

The present study was a randomized controlled clinical trial in which 70 family caregivers of people suffering from dementia were sampled through the block method and they were divided into experimental and control groups. The data collection tools included demographic information questionnaire and grief inventory that was completed before, a week and a month after the implementation of the compassion-based program in five sessions virtually by sending offline content to the experimental group. The obtained data were analyzed by SPSS 22 software using the repeated measures analysis of variance.

**Results:**

The mean comparison of scores of the grief experienced in the experimental group between pretest and posttest and follow-up, unlike the control group, showed a significant difference (*p* < 0.001). The mean scores of the experimental and the control groups were significantly different in terms of experiencing mourning and all of its fields (*p* < 0.001). The mean comparison of post-test and follow-up showed that the effect of a compassion-based program at the follow-up stage has been mitigated.

**Conclusions:**

According to the study results, the compassion-based program reduced the grief experienced by the family caregivers of people suffering from dementia. Nevertheless, the effect of a compassion-based program has been reduced over time. This program can be a good guide for providing society-oriented services to the health team.

**Trial registration:**

This study is registered by Iranian Registry of Clinical Trials with decree code:IRCT20190712044181N4 on 02/06/2020.

## Background

Dementia is a progressive brain disorder [1]. According to the World Health Organization (WHO), in 2016, approximately, 47 million people around the world were diagnosed with dementia, and this figure by 2030 will be increased to more than 75 million people and by 2050, their population will almost be three times. As the disease progresses and the abilities of people suffering from dementia reduce, the need for care of these patients increases [2].

Care by definition is considering other people’s health needs. In general, care is divided into two categories of “formal care” and “informal care”. Informal caregivers (primary/unpaid) have a variety of titles and labels, such as non-specialist, untrained, and family caregivers [3]. Family caregivers are those who are not paid for providing the care [4]. They usually help meet basic day-to-day functional needs and play a major role in providing care for the dependent patient [5]. One of the most important policies of the health care system in the world is to increase and support care programs for people suffering from dementia at home by their families [6].

Providing people suffering from dementia with care involves unique challenges. For instance, at the middle stages of the diseases, these patients suffer from deterioration in judgment and orientation, and their ability to comprehend and start an effective communication is hampered. Caregivers are required to deal with these complications and help the patients. Besides, this disease causes the personality and behavior of the patient to change inflicting more challenges to the caregivers [7]. As the disease symptoms worsen, emotional stress and depression are intensified leading to worsened health problems as well as the rise of their new types, and the financial and economic costs of the caregiver are exacerbated and their job and payment for the related services and their health care or the patient’s care experience disruption [8].

A larger share of care for people suffering from dementia is provided by family caregivers and due to the great care burden, the physical and mental health of family caregivers is damaged [9]. However, family caregivers experience positive emotions such as family solidarity and satisfaction with helping others in the care process [10]. Family caregivers of people suffering from dementia have twice as many financial, emotional, and physical problems as family caregivers of those without dementia [11]. Finally, a set of pressures that the patients care creates for family caregivers reduces the quality of care for the patient, resulting in deteriorating patients’ health and worsening the behavioral and psychological symptoms of dementia [12], leading to the decision for transferring the patient to care centers [13], reducing the quality of life of family caregivers and inflicting destructive and negative impacts on family caregivers’ health and different degrees of grief experience [14]. Grief is a reaction to the perception of loss [15].

As the dependency theory stipulates, grief is experienced by the caregivers of people suffering from dementia due to the lost feeling of attachment and dependency caused by hampered cognition and progressive memory absence. As the extent of former attachment grows, the feeling of grief and loss is intensified [14].

Grief experience also varies depending on the relationship between caregiver and patient and the stage of the disease [16].

Grief of these caregivers implies twofold death since their grief is first experienced prior to the actual death of the patient due to lost attachment and feeling of dependency caused by the mitigated progressive memory loss of the patient; then, the grief is experienced once more following the real death of patient [14].

Its common symptoms include sadness, regret, guilt, emotional distress, anxiety, loneliness, and anger, and it is an initial reaction in family caregivers of people suffering from dementia [14].

Finally, these care complications and negative responses and emotions lead to the psychological manifestations of despair, lack of interest in doing things, community withdrawal, and a reduction or an increase in dependence [17]. Withdrawal from the patient, dependence reduction and involvement of family caregiver in these complications and pressures can reduce the quality of care [12] and affect the necessary attention and concern towards the patient; vice versa, fear of empathy and lack of compassion can also predispose the family caregiver to depression, anxiety, stress and grief symptoms and intensify the destructive effects of care. Thus, compassion can be affected by negative psychological responses, and lack of compassion can exacerbate these responses.

Numerous interventions have been proposed to assist the family caregivers of people suffering from dementia. All of these interventions aim to boost these family caregivers’ health and well-being, mitigate the undesirable characteristics of care, delay the admission of the individuals suffering from dementia to care centers, assist the caregivers in dealing with symptoms related to dementia, promote community support for caregivers, and provide opportunities for them to implement their responsibilities. Counseling, support groups, psychotherapy and psychological methodologies are among those interventions [11]. Compassion-based interventions are a branch of psychological interventions implemented to boost individuals in terms of their physical and mental conditions by compassion promotion [18].

Different definitions of compassion have been proposed. Goetz et al. considered compassion as sensitivity to the perception of suffering and a desire to alleviate it [19]. Compassion plays an important role in emotion regulation and can increase patience to weaknesses of other human beings and improve the health and well-being of the mind [18, 20]. Compassion is a concept that can be taught to individuals [21, 22]; it can be practiced, strengthened and expanded [23] leading to the emergence of compassion-based therapies. Compassion has been used by various researchers in a variety of ways, including in-person [24–26] or not in-person [27, 28]. Nevertheless, for the first time, Gilbert, in his study and during his patients’ treatment sessions, introduced the theory of compassion-based therapy [29]. The main principles of this theory are based on the fact that the external relieving factors can be transformed internally leading to the peace of mind [30, 31]. Compassion-based programs facilitates the appropriate response of individuals to negative events in their life [32]. Since some studies have shown that the effect of compassion-based program has different effects on different variables [24–26] and no study has been conducted on family caregivers of people suffering from dementia in Iran in this regard, the researchers conducted this study to investigate the effect of a compassion-based program on the grief experienced by caregivers of people suffering from dementia.

## Methods

The present study is a clinical randomized controlled trial (RCT) that was recorded with code of ethics IR.MUI.RESEARCH.REC.1399.076 and code of clinical trial IRCT2019712044181N4 on 02/06/2020; it was designed to investigate the effect of a compassion-based program on the experienced grief of caregivers of people suffering from dementia.

### Information of the participants

In this study, using the formula $=\frac{{({z}_{1}+{z}_{2})}^{2}{(2s)}^{2}}{{d}^{2}}$, at least, 32 people were calculated and 35 people were selected in each group, given the 10% sample drop. Z_1_ at 95% confidence level was 1.96. Z_2_ of test power factor of 80% was equal to 0.84. S is an estimate of the mean standard deviation of the grief experience score of family caregivers of people suffering from dementia in the two experimental and control groups, and D is the minimum difference between the mean grief experience score of family caregivers of people suffering from dementia between the experimental and control groups, indicating a significant difference. S was considered 0.7. A total of 70 family caregivers were selected from two clinics (Clinics of Amin Hospital and Khorshid Hospital) with inclusion criteria as follows: reading and writing literacy, willingness to participate in a compassion-based program, if the patient has multiple family caregivers, the caregiver who spends more time with the patient was included, access to cyberspace to receive the content of the compassion-based program and family caregivers whose patient was at the first or second stage of the disease with uncontrolled diabetes, cancer, and no history of stroke.

### Processes and interventions

First, the data of family caregivers of people suffering from dementia were extracted through their records in medical centers by obtaining a permission on condition of complete anonymity. Then, the family members were contacted and they were informed about the objectives and the researchers of this study considered their willingness to participate in this study. Sampling was performed using the blocking method. Six blocks consisted of four people, from whom two family caregivers were randomly selected)by drawing lots by writing on paper( as samples of the control group and two family caregivers were chosen as samples of the experimental group. Finally, 70 family caregivers of people suffering from dementia were included in the study. After completing the samples of each block, the program was run separately from the other blocks. The research was continued in each block in such a way that with the participant’s consent to participate in the study, the participant determined the appropriate date and time for the researcher to contact him and the participant’s contact number was provided to the family caregivers and the collected data were transferred to him by the center personnel.

After randomly assigning the samples to the two groups, at the time determined by the family caregiver, the researcher contacted the family caregivers to familiarize them with the project and fully inform them about the project implementation process and provided them with the necessary explanations. Then, both experimental and control groups received and completed the electronic form of consent, demographic information questionnaires and grief experience inventory.

Family caregivers of the experimental group were informed that the virtual sessions of the compassion-based program would begin a week later. The researcher’s phone number was also provided to the family caregivers so that they could ask any questions. Then, five not in-person sessions of the compassion-based program were held weekly for the members of the experimental group. The content of the sessions was prepared and presented by the nurse as the organizer and under the supervision of a psychiatrist. Each week, the content prepared for each session was sent to the family caregivers in the form of multimedia in cyberspace and through the WhatsApp application. It should be noted that at the beginning of each session, the content of the previous session was reviewed, and the family caregivers talked about their experiences of the past week in relation to compassion. At the end, the assignment was sent to family caregivers with a training booklet (Word file). Family caregivers reported weekly on their assignment to the researcher via WhatsApp. During the week, the samples were given the opportunity to ask questions, comment or express their experiences through the contact number of the nurse. Thus, family caregivers enjoyed the presence of the nurse and interaction with him during the week.

The content of the first session of the compassion-based program for the experimental group included establishing initial communication with family caregivers, familiarizing them with the principles of the compassion-based program, and conceptualizing compassion by the nurse. In the next session of mindfulness training, body examination and breathing exercises were performed virtually by the nurse for the experimental group in a simple and understandable manner, and instructional videos were provided to the family caregivers. The third session of the compassion-based program was conducted to familiarize them with the characteristics of compassion and the fourth session was conducted to train compassion skills to the experimental group. In the following week, in the fifth session of the program, the experimental group received training based on compassion, acceptance of important problems and events, confronting various challenges and writing letters.

After completing the compassion-based program in the experimental group, participants in both the experimental and control groups were asked to complete grief experience inventory electronically again. Thus, one week after the implementation of the compassion-based program, the above questionnaire was completed by the members of both groups. In order to follow the long-term effects of the program, one month after the implementation of the compassion-based program, grief experience inventory was completed again by both groups. Then, for ethical considerations, after completing the questionnaire and implementing the compassion-based program, the content in the form of files and videos along with educational booklets was provided to the control group (Table 1).

**Table 1:** Summary of sessions of the compassion-based program

Session	Content
1	establishing initial communication with caregivers, familiarizing them with the principles of compassion-based program and understanding compassion
2	mindfulness training, body checking and breathing exercises
3	familiarizing with the principles of compassion
4	training compassion skills
5	training to accept problems and upcoming events, face different challenges and write letters

### Data collection tools

The demographic information questionnaire included information about age, gender, education, occupation, marital status, and care characteristics.

Marwit and Meuser Caregiver Grief Inventory (MM—CGI—SF) was used to measure grief experience. This tool is the short form consisting of eighteen questions that investigate the degree of grief of caregivers of people suffering from dementia in three fields of personal sacrifice burden, heartfelt sadness and longing, and worry and isolation. Each field covers six of the eighteen questions in the questionnaire. Each question is scored from one to five. Each score represents a specific concept (1 = strongly disagree, 2 = disagree, 3 = somewhat agree, 4 = agree, and 5 = strongly agree). In the first field, the range of scores varies from 1 to 30, and if a score of 21 or more is obtained, it indicates the experience of great personal sacrifice burden. In the second field, the range of scores varies from 1 to 30, and a score of 21 or more indicates great heartfelt sadness and longing. In addition, in the third field, the range of scores varies from 1 to 30, and a score of more than 17 indicates great worry and isolation. Finally, according to the sum of the scores obtained from the three fields, the general level of grief is obtained, which varies from a score of 1 to 90 and a score of 57 is the average level of grief experience and a standard deviation higher than that indicates a high level of grief experience and a standard deviation lower than that indicates low grief experience [33].

Sanders et al. applied and validated this questionnaire in 2008. Cronbach’s alpha was determined to be in the three areas of care burden due to the personal sacrifice (alpha = 0.83), the area of heartfelt sadness and longing (alpha = 0.84) and the area of great worry and isolation (alpha = 0.78) [14].

### Statistical analysis

Finally, the collected data were extracted from the questionnaires and analyzed by SPSS 22 software using the repeated measures analysis of variance.

## Results

According to the results of the Tables 2, 77% of the experimental and 88% of control groups participants were female. 65% of experimental group and 91% of control group were married. 48% of the experimental and 54% of control groups were housewives. Only 5.7% of the participants had a history of patient care. 85% family caregivers in experimental and 94% in control groups were satisfied with their patient care (Tables 2).

**Table 2:** Demographic information of the statistical sample

Variable	Level	Experimental group	Control group	Chi-square test	
		F	F	Chi-square test	Sig
**Gender**	female	27	31	0.32	0.58
	male	8	4		
**Marital status**	widow	2	2	0.15	0.66
	married	26	31		
	single	5	1		
	divorced	2	1		
**Level of education**	High school	8	6	0.27	0.68
	Diploma	13	15		
	B.A	14	14		
**Employment status**	retired	0	2	0.19	0.72
	unemployed	2	2		
	housewife	14	20		
	Self-employed	7	6		
	Employee and worker	10	5		
**Satisfaction with care**	+	30	33	0.97	0.11
	-	5	2		
**Previous patient care training history**	+	1	2	0.13	0.44
	-	34	33		

The mean age of experimental participants was 45.48 years and the mean age of control participants was 43.35 years. The mean duration of care for an individual suffering from dementia of the experimental group was 2.5 years and the mean duration of care for an individual suffering from dementia of control group was 2.6 years. The mean hours of daily patient care of experimental group was 12.40 h and the mean hours of daily patient care of control group was 13.10 h (Table 3). As Tables 2 and 3 show, the information of the sample in the two groups is not significantly different.

**Table 3:** Comparison of mean age and duration of the patient care

Variable	Experimental group		Control group		t-test	
	M	SD	M	SD	t	Sig
**Age**	45.48	17.36	43.35	8.56	-0.998	0.322
**Duration of care for a** people suffering from dementia **(years)**	2.5	0.754	02.6	0.812	0.817	0.456
**Hours of daily patient care**	12.40	1.145	13.10	2.17	1.285	0.098

Table 4 shows that grief experience score and the components of the two groups in the pre-test are close to each other. In addition, these results showed that at the post-test and follow-up stages, the score of the experimental group reduced and the score of the control group did not change significantly. Therefore, it is necessary to investigate whether the intervention reduced the mean in the experimental group or whether this reduction in the mean is due to other intervention factors. According to the results, the main effect of group (*p* < 0.001) and the main effect of time (*p* < 0.001) as well as the interaction between the group and time (*p* < 0.001) were statistically significant. The effect size of the group showed that 23% of the changes in the mean grief experience were due to the effect of the group, 43% of the changes were due to the effect of time, and finally 58% of the changes were due to the interaction between the group and time. Therefore, the mean of the experimental group is significantly different from the control group. Due to the significant effect of time, to determine if the difference between the mean of pretest, posttest and follow-up in which of the three stages was significant, Bonferroni post hoc test was used; the results showed that in the experimental group, a significant difference was in the test and follow-up of grief experience (*p* < 0.001). In addition, a significant difference was in the mean of post-test and follow-up (*p* < 0.001) (Table 4).

**Table 4:** Descriptive indicators of grief experience

**Variable**		**Group**	**M**	**SD**	**Independent t-test**	
					T	Sig
**Grief experience**	pre-test	Experimental group	71.34	6.95	1.133	0.266
		Control group	69.14	9.14		
	post-test	Experimental group	56.82	6.93	-6.921	> 0.001
		Control group	70.74	9.66		
	follow-up	Experimental group	60.60	6.00	-7.168	> 0.001
		Control group	72.42	7.67		
**Frequent measurement variance**	Control group	F	0.459			
		P	0.503			
	Experimental group	F	32.390			
		P	> 0.001			

In all fields of grief, the results showed that the main effect of group (*p* < 0.001) and the main effect of time (*p* < 0.001) as well as the interaction between the group and time (*p* < 0.001) were statistically significant. The effect size of the group showed that in the field of ​​ personal sacrifice burden, 23% of the average changes were due to the group effect, 16% of the changes were due to the effect of time and finally 38% of the changes were due to the interaction between the group and time (Table 5).

**Table 5:** .Descriptive personal sacrifice burden component of grief

**Variable**		**Group**	**M**	**SD**	**Independent t-test**	
					T	Sig
**Personal sacrifice burden**	pre-test	Experimental group	23.05	3.40	0.715	0.477
		Control group	22.45	3.61		
	post-test	Experimental group	18.71	2.91	-5.714	> 0.001
		Control group	23.17	3.57		
	follow-up	Experimental group	20.02	2.39	-5.434	> 0.001
		Control group	23.48	2.90		
**Repeated measurement variance**	Control group	f	6.531			
		p	0.015			
	Experimental group	f	11.240			
		P	> 0.001			

In the field of heartfelt sadness and longing, 11% of the changes were due to the group effect, 31% of the changes were due to the time effect and finally 44% of the changes were due to the interaction between the group and time (Table 6).

**Table 6:** Descriptive heartfelt sadness and longing component of grief

**Variable**		**Group**	**M**	**SD**	**Independent t-test**	
					T	Sig
**Heartfelt sadness and longing**	pre-test	Experimental group	25.51	3.05	1.611	0.112
		Control group	24.11	4.13		
	post-test	Experimental group	20.25	2.95	-4.612	> 0.001
		Control group	24.57	4.67		
	follow-up	Experimental group	21.68	2.69	-5.194	> 0.001
		Control group	25.57	3.50		
**Repeated measurement variance**	Control group	F	0.37			
		P	0.848			
	Experimental group	F	39.435			
		P	> 0.001			

In the field of worry and isolation, 30% of the changes were due to group effect, 38% of the changes were due to the time effect and finally 50% of the changes were due to the group and time interaction. The results of post hoc test in all fields of grief showed a significant difference between post-test and follow-up (*p* < 0.001). In addition, a significant difference was in the mean of post-test and follow-up (*p* < 0.001) (Table 7).

**Table 7:** .Descriptive worry and felt Isolation component of grief

**Variable**		**Group**	**M**	**SD**	**Independent t-test**	
					T	Sig
**Worry and felt Isolation**	Pre-test	Experimental group	22.77	2.47	0.288	0.774
		Control group	22.57	3.27		
	Post-rest	Experimental group	17.85	2.35	-7.948	> 0.001
		Control group	23.00	3.01		
	Follow-up	Experimental group	18.91	2.21	-7.390	> 0.001
		Control group	23.37	2.79		
**repeated measurement variance**	Control group	F	0.104			
		P	0.750			
	Experimental group	F	2.927			
		P	> 0.001			

## Discussion

The objective of this study was to investigate the effect of a compassion-based program on the grief experienced by caregivers of people suffering from dementia. The study results showed the effectiveness of the compassion-based program on reducing grief experienced by this group of family caregivers in the post-test and follow-up in the experimental group was contrary to the control group. In addition, in all three fields of the grief experience (personal sacrifice burden, heartfelt sadness and longing, and worry and isolation) in the experimental group, unlike the control group, a significant reduction was observed, indicating the effect of the compassion-based program on the grief experience by family caregivers in all fields. Parallel to this finding, a study conducted by Poz (2018) has shown that the use of compassion-based programs for those who have recently been diagnosed with dementia can also be effective [34]. A clinical trial by Meichsner (2016) investigated the cognitive-behavioral intervention on the grief experienced by 33 family caregivers of people suffering from dementia also showed the effect of cognitive-behavioral intervention on the grief experience of family caregivers. This study is a comprehensive intervention focusing on strengthening the mind and accepting the problem with four categories of intervention; cognition and accepting loss and change, normalizing grief, meaning and redefining communication in new situations, addressing future and future loss could reduce the grief experienced by family caregivers of people suffering from dementia. The study by Meichsner, like the present study, used not in-person (telephone) method that allowed the samples to participate with peace of mind. However, the study by Meichsner differs from the present study due to the greater number of test sessions and qualitative analysis of data [27].

Lena Axelsson et al. (2020) in a study entitled “grief intensity prior to and following the death experienced by family caregivers in palliative care regarding the preparation for care, caregivers’ care burden, and community support” reported that caregivers with greater documented care burden experienced greater grief before death [35]. This study applied the Grief Experience Questionnaire according to which the average care scores higher 21 showed greater care burden in these individuals. The mean scores of care burden in the experimental group in the pre-test, in the post-test, and the follow-up stages were 23.05, 18.71, and 20.02, respectively, showing that the caregivers encountered great care burden at first and this burden was mitigated through the application of the compassion-based program. Earlier studies have shown that great care burden of family caregivers leads to their deteriorated physical and mental health [9] and reduced quality of life [4] imposing complications like heart disease and kidney disorders on them [36–38]. Tau Ming Liew et al. (2017) compared the long-term impacts of grief and care burden on the depression of caregivers of people suffering from dementia and they reported that caregivers experiencing greater grief and care burden show intensified symptoms of depression in comparison with other caregivers [39]. Accordingly, improvising interventions like compassion-based programs to mitigate the effects of these complications along with alleviating the care burden of family caregivers seems highly important.

Our findings reveal that heartfelt sadness and longing of the family caregivers of people suffering from dementia have been affected by the compassion-based program and the intensity of heartfelt sadness and longing of the experimental group compared to control group has been mitigated in the post-test and follow-up periods. Aslani and Eskandari (2017) reported that enhanced compassion could affect negative emotions [20]. Shariati et al. (2015) argued that depression as a set of negative emotions can be mitigated in female university students through compassion-based treatment [40]. Since the heartfelt sadness and longing seem to be negative emotions, these findings are consistent with results of the present study.

Moreover, our findings showed that the feeling of worry and isolation of the family caregivers of people suffering from dementia has been affected by the compassion-based program and the intensity of the feeling of worry and isolation in the experimental group compared with the control group has been reduced at the post-test and follow-up stages. Navab et al. (1390) reported that intensified feeling of isolation in this group of family caregivers could be due to fact that the caregivers are engaged in caring of the patients and as a result their effective communications with friends and relatives is reduced and they are sometimes forced to be deprived of professional and social activities. Thus, their material and mental support is reduced and the feeling of isolation is intensified [12] and these factors have been justified in the present study.

For the long-term effects of the compassion-based program on family caregivers’ grief experience, the present results showed that although the average grief experience in the experimental group, unlike the control group, reduced in follow-up compared to the pre-test, comparing the mean of post-test and follow-up showed that effects of the compassion-based program at the follow-up stage have been reduced compared to the post-test regarding the grief experience and all its fields. The results of the present study are consistent with a study by Ghaffarian and Khayatan (2015) entitled “effects of compassion-based therapy on self-concept and self-expression of female high school students” which showed the effect of compassion-based therapy on their expression was significant only in the post-test stage, but this effect was not significant in the one-month follow-up and had no significant effect on the self-concept of female students [41]. Unlike the present study, in a study by Shariati et al. (2016), compassion-based therapy had an effect on reducing students’ depression and self-criticism and this effect continued until the follow-up period [40]. In a study by Wagner et al. (2006), the effects of cognitive-behavioral intervention on grief were maintained until three months after the intervention [28]. According to the above results, reducing the effects of the program in the long time can be due to various factors that need further studies.

### Limitations

In this study, there were limitations such as unclear mental and physical health of the samples when completing the questionnaires and the limited sample group to a province. Therefore, due to the difference in the culture regarding the understanding of concepts such as compassion, generalizing the study results should be done with caution.

### Suggestions

Since the compassion-based program in the present study had a significant effect on the existing content and structure, researchers can conduct further studies to alleviate grief experience of this group of family caregivers. Clinically, family caregivers can be examined for grief experience from the time of admission of people suffering from dementia in care centers or related communities, and if there is a problem, they can be helped using interventions such as the compassion-based program. This intervention for family caregivers of people suffering from dementia or other family caregivers of people with other chronic diseases can reduce care complications of family caregivers, increase the quality of care provided to patients and reduce treatment-care costs.

## Conclusion

Finally, the results of the present study showed that the compassion-based program could improve grief experience in all fields for family caregivers of people suffering from dementia. In addition, according to the study results, the effects of the compassion-based program have been reducing after 1 month, so the long-term effects of this program should be further studied.

## Data Availability

The datasets generated and analyzed during the current study are not publicly available due to the identity information contained in the data but they can be obtained from the corresponding author on reasonable request by deleting this information.

## Abbreviations

RCT: Randomized controlled Trial
MM – CGI – SF: Marwit and Meuser Caregiver Grief Inventory, Short Form
CBT: Cognitive Behavioral Therapy
